# Nanostructured Lipid Carriers Enable In Vivo Efficacy of Parthenolide in *Schistosoma mansoni* Infection

**DOI:** 10.3390/pharmaceutics18060694

**Published:** 2026-06-03

**Authors:** José Márcio Fernandes da Silva, Dominique Mesquita e Silva, Danilo de Souza Costa, Monique C. Amaro, Rayssa A. Cajas, Josué de Moraes, Guilherme Diniz Tavares, Ademar Alves Da Silva Filho

**Affiliations:** 1 Departamento de Ciências Farmacêuticas, Faculdade de Farmácia, Universidade Federal de Juiz de Fora, R. José Lourenço Kelmer s/n, Campus Universitário, Juiz de Fora 36036-900, MG, Brazil; jmf_farm@yahoo.com.br (J.M.F.d.S.); dominiquefarmacia@gmail.com (D.M.e.S.); scdanilo@gmail.com (D.d.S.C.); guilherme.tavares@ufjf.br (G.D.T.); 2 Núcleo de Pesquisa em Doenças Negligenciadas, Universidade Guarulhos, Guarulhos 07023-070, SP, Brazil; moniqueamaronpdn@gmail.com (M.C.A.); rayssacajas@gmail.com (R.A.C.); moraesnpdn@gmail.com (J.d.M.); 3 Núcleo de Pesquisa em Doenças Negligenciadas, Instituto Científico e Tecnológico, Universidade Brasil, São Paulo 08230-030, SP, Brazil

**Keywords:** *Schistosoma mansoni*, Parthenolide, Nanotechnology, Nanoparticles

## Abstract

**Background:** Schistosomiasis remains a major neglected tropical disease, with praziquantel (PZQ) as the only widely used treatment, despite its limitations. Parthenolide (PTL), a sesquiterpene lactone, exhibits potent in vitro antischistosomal activity; however, its poor aqueous solubility, low oral bioavailability, and chemical instability may limit its in vivo efficacy. **Objective:** This study investigated whether nanoencapsulation in nanostructured lipid carriers (NLC) could enable the in vivo antischistosomal activity of PTL. **Methods:** PTL was isolated from *Tanacetum parthenium* and incorporated into NLC using hot emulsification followed by ultrasonication. The resulting formulation (NLC-PTL) was physicochemically characterized, and its in vivo antischistosomal efficacy was evaluated in a murine model of *Schistosoma mansoni* infection. **Results:** NLC-PTL exhibited nanoscale size, low polydispersity, high encapsulation efficiency, and sustained drug release. In vivo, free PTL showed no significant effect on worm burden, whereas NLC-PTL achieved a marked reduction (77.9%) in adult worms and significantly decreased egg output compared to controls (*p* < 0.001). Blank NLC had no antiparasitic effect. **Conclusions:** Nanoencapsulation was associated with in vivo antischistosomal activity of PTL compared to the free compound. These findings suggest that formulation strategies may influence the in vivo performance of lipophilic natural products in schistosomiasis.

## 1. Introduction

Schistosomiasis is a neglected tropical disease caused by blood-dwelling parasitic flatworms of the *Schistosoma* genus, mainly *S. mansoni*, and remains a major public health challenge despite decades of control efforts [1]. It is estimated that nearly 250 million people are affected annually across 80 countries, with a substantial impact on quality of life in endemic populations [2,3].

Sustained transmission persists in various endemic areas, particularly in South America, the Middle East, and sub-Saharan Africa, where over 90% of cases are concentrated, despite ongoing mass drug administration and prevention campaigns [2].

Praziquantel (PZQ) remains the cornerstone of schistosomiasis treatment; however, its use is limited by several drawbacks. These include the need for relatively high therapeutic doses, largely due to its low aqueous solubility and variable oral bioavailability [4], as well as concerns regarding reduced drug sensitivity in certain endemic areas [5]. Together, these limitations highlight the urgent need for new antischistosomal agents and, importantly, for alternative strategies capable of improving the pharmacological performance of existing or emerging compounds [6,7,8].

In this context, natural products have emerged as a valuable source of structurally diverse molecules with multitarget antiparasitic activity [9,10]. Plant-derived molecules may act through various mechanisms, including tegumental damage, impaired motility, and interference with metabolic pathways [10,11].

Among them, parthenolide (PTL), a sesquiterpene lactone isolated from *Tanacetum parthenium* (feverfew), has attracted attention due to its broad biological profile, including antitumor, anti-inflammatory, and antiparasitic effects [12,13,14,15]. In vitro studies have demonstrated that PTL induces severe tegumental damage and parasite death at micromolar concentrations, supporting its potential as an antischistosomal candidate [14,16]. However, despite this promising activity, the in vivo efficacy of PTL is expected to be limited by its unfavorable physicochemical properties, including poor aqueous solubility, low oral bioavailability, and chemical instability [17,18].

To overcome these biopharmaceutical limitations, nanoscale drug delivery systems have been increasingly explored [11,19,20]. Nanostructured lipid carriers (NLC), in particular, represent a second-generation lipid-based system composed of a mixture of solid and liquid lipids, resulting in a less ordered internal structure that enhances drug loading and retention [21,22]. These systems can improve the stability, absorption, and controlled release of lipophilic compounds, thereby increasing their therapeutic potential [23,24]. Indeed, growing evidence indicates that NLC formulations can significantly enhance the in vivo performance of poorly soluble natural products [25,26].

Based on this rationale, the present study aimed to investigate whether nanoencapsulation could enable the in vivo antischistosomal activity of PTL. To this end, PTL was isolated from *T. parthenium*, incorporated into NLC, and the resulting formulation was physicochemically characterized and evaluated in a murine model of *S. mansoni* infection.

## 2. Materials and Methods

### 2.1. Chemicals and Reagents

HPLC-grade methanol and acetonitrile were purchased from Merck (Darmstadt, Germany). Formic acid and phosphoric acid of HPLC quality were supplied by Aladdin Industrial Corporation (Shanghai, China). Praziquantel (PZQ) was kindly provided by Ecovet Indústria Veterinária Ltd. (São Paulo, SP, Brazil). RPMI 1640 culture medium and heat-inactivated fetal bovine serum were purchased from Vitrocell (Campinas, SP, Brazil). Reagents including HEPES buffer, glutaraldehyde, and dimethyl sulfoxide (DMSO) were sourced from Sigma-Aldrich (St. Louis, MO, USA). *Passiflora edulis* seed butter was acquired from Ebpm Comercial Ltd. (São Paulo, SP, Brazil), while Miglyol^®^ 812 was obtained from Engenharia das Essências (São Paulo, SP, Brazil). All other reagents and solvents employed were of analytical grade.

### 2.2. Isolation and Purification of Parthenolide

Inflorescences of *T. parthenium* were collected near the Faculty of Pharmacy, at the Federal University of Juiz de Fora (UFJF), Brazil (21°46′34.8″ S, 43°21′52.4″ W). A voucher specimen (CESJ 65105) was deposited at the Leopoldo Krieger Herbarium, Institute of Biological Sciences, UFJF. The study was conducted in accordance with Brazilian Federal Law No. 13.123/2015, which regulates access to genetic resources (Sisgen #ADE4875).

The inflorescences of *T. parthenium* (700 g) were dried at 40 °C, powdered, and extracted, by maceration, with ethyl acetate. The solvent was evaporated under reduced pressure to yield the crude extract (TpE) (63.1 g). The crude extract (TpE, 60 g) was subjected to low-pressure liquid chromatography over silica gel (70–230 mesh). Elution was carried out using mixtures of hexane and ethyl acetate, yielding six pooled fractions (Fr I–VI). Fraction IV (10.2 g) was further purified by column chromatography employing a hexane: ethyl acetate (7:3, *v*/*v*) solvent system, which afforded a white solid (5.1 g). The isolated compound was identified as parthenolide based on ^1^H and ^13^C NMR spectroscopic data (See Supplementary Figures S1 and S2).

### 2.3. HPLC Analysis of PTL

The isolated PTL was characterized using high-performance liquid chromatography coupled with diode array detection (HPLC-DAD) on a system manufactured by Waters Corporation (Milford, MA, USA). Separation was performed on a SunFire C_18_ column (5 µm, 4.6 × 250 mm; Waters). The mobile phase consisted of ultrapure water containing 0.1% acetic acid (phase A) and methanol of HPLC grade (phase B). Elution was carried out under gradient conditions, starting with 60% A and 40% B for 40 min, followed by a transition to 100% B from 40 to 45 min. The flow rate was kept constant at 1.0 mL/min during the entire run.

### 2.4. Development and Characterization of Parthenolide-Loaded Nanoparticles

#### 2.4.1. Preparation and Evaluation of Nanostructured Lipid Carriers Containing Parthenolide

NLC were prepared using the hot emulsification method followed by ultrasonication, as previously described by Mall et al. [27], with some adaptations. Briefly, the lipid phase consisted of *Passiflora edulis* seed butter (PB, 3.9% *w*/*v*), Miglyol^®^ 812 (M812, 3.0% *w*/*v*), and PTL (0.1% *w*/*v*), while the aqueous phase comprised an aqueous dispersion of Pluronic™ F-127 (PF-127, 1.8% *w*/*v*), as detailed in Table 1. Both phases were heated separately to 10 °C above the lipid melting point (55 °C). The aqueous phase was then added to the molten lipid phase under probe sonication (70% amplitude) for 3 min, followed by an additional 3 min of sonication in an ice bath. For comparison purposes, drug-free NLC (NLC-B) were prepared using the same procedure. The resulting nanodispersions were stored under refrigeration (4 °C) until further use.

#### 2.4.2. Evaluation of Particle Size, Polydispersity Index, and Zeta Potential

The particle size (PS) and polydispersity index (PdI) of the nanoparticles were determined by dynamic light scattering (DLS) using a detection angle of 173° and a refractive index of 1.333. The zeta potential (ZP) was obtained from the electrophoretic mobility of the particles. Prior to analysis, samples were diluted at a ratio of 1:800 (*v*/*v*) in ultrapure water produced using a Direct-Q^®^ 3 UV water purification system (Merck Millipore, Darmstadt, Germany). Measurements were performed at room temperature in triplicate using a Zetasizer Nano ZS90 (Malvern^®^, Worcestershire, UK) [28,29].

#### 2.4.3. pH

The pH of the formulations (NLC-B and NLC-PTL; *n* = 3) was measured directly in samples maintained at room temperature. Measurements were performed using a portable pH meter (MPA210P, Tecnopom^®^, São Paulo, Brazil), previously calibrated with standard buffer solutions at pH 4.0, 7.0, and 10.0.

#### 2.4.4. Encapsulation Efficiency

The encapsulation efficiency (EE%) of PTL in the nanoparticles was determined indirectly by the ultrafiltration–centrifugation method, as previously described [28], with methodological adaptations. Briefly, samples were subjected to centrifugation at 4.000 rpm for 30 min using Amicon^®^ ultrafiltration units (MWCO 10 kDa; Millipore, Germany). The ultrafiltrate, containing the non-encapsulated fraction of PTL, was collected and quantified by HPLC-DAD at a detection wavelength of 215 nm [29]. Chromatographic analysis was carried out using a C_18_ column (5 μm particle size, 4.6 × 250 mm) under isocratic conditions, with a mobile phase consisting of methanol/water (65:35, *v*/*v*) containing 0.1% acetic acid, at 40 °C and a flow rate of 1 mL/min. The EE (%) was calculated based on the difference between the total amount of PTL added and the free fraction quantified in the ultrafiltrate.

(1) $$EE\;\left(\%\right)=\frac{Total\;drug\;added-amount\;of\;drug\;quantified\;}{Total\;drug\;added}\times 100$$

#### 2.4.5. In Vitro Release Studies

The in vitro release of PTL was evaluated using the dialysis bag method at 37 °C under constant agitation (70 rpm). A 0.9% (*w*/*v*) sodium chloride solution containing 10% (*v*/*v*) ethanol was used as the receptor medium to maintain sink conditions [30]. The release studies were conducted in two different media: hydrochloric acid solution (pH 1.2), simulating gastric conditions, and phosphate buffer (pH 6.8), representing the intestinal environment. Samples of NLC-PTL were placed into dialysis bags (Servapor^®^, MWCO 12.000–14.000), previously hydrated and properly sealed, and then immersed in the receptor medium. At predetermined time intervals, aliquots of the receptor solution were withdrawn and immediately replaced with fresh medium to maintain constant volume. Sampling was performed at 0, 1, 2, 4, 8, and 12 h for pH 6.8, and at 0, 15, 30, 60, and 120 min for pH 1.2. The amount of PTL released was quantified by HPLC-UV-DAD at a detection wavelength of 215 nm. The cumulative amount of PTL released was plotted as a function of time to obtain the release profile.

The release mechanism of PTL from the nanoparticles was investigated by fitting the experimental data to the main kinetic models described in the literature, including zero-order, first-order, Higuchi, and Korsmeyer–Peppas models [31]. Mathematical modeling was performed using Microsoft Excel with the DDSolver add-in, a widely used tool for quantitative analysis of drug release profiles. The goodness of fit of each model was evaluated based on the coefficient of determination (R^2^) and the Akaike Information Criterion (AIC), allowing the identification of the model that best described the experimental data. Additionally, for the Korsmeyer–Peppas model, the release exponent (*n*) was determined to elucidate the predominant release mechanism, considering contributions from diffusion, matrix relaxation, or a combination of both processes.

#### 2.4.6. Colloidal Stability Studies

The colloidal stability of NLC-PTL was evaluated during storage at 4 °C over a period of 90 days, with analyses performed at 0, 30, 60, and 90 days, as described by Silva [30]. Throughout the study, changes in PS, PdI, and ZP were monitored to assess the physical stability of the formulations.

### 2.5. In Vivo Antischistosomal Studies

#### 2.5.1. Animals and Parasite Maintenance

All experiments were conducted using the BH strain of *S. mansoni*, which was continuously maintained under laboratory conditions through its biological cycle involving *Biomphalaria glabrata* snails as intermediate hosts and Swiss mice as definitive hosts. The parasite life cycle was sustained at the Research Center on Neglected Diseases, Guarulhos University (São Paulo, Brazil) [32]. Female Swiss mice (approximately 25 g, 3 weeks old) were obtained from Anilab (São Paulo, Brazil) and housed under standardized environmental conditions (25 °C, 50% relative humidity, 12 h light/dark cycle), with unrestricted access to food and water. To maintain the parasite cycle, mice were subcutaneously inoculated with 100 *S. mansoni* cercariae per animal, following established protocols [10,30,33]. This higher inoculum is routinely used for parasite maintenance purposes and differs from the infection load employed in therapeutic efficacy studies.

#### 2.5.2. In Vivo Antischistosomal Evaluation

Female mice were infected subcutaneously with 80 *S. mansoni* cercariae according to established protocols [10,30,34]. This inoculum was selected to establish a patent infection while minimizing excessive morbidity, as commonly adopted in in vivo antischistosomal efficacy studies [35]. At 42 days post-infection (corresponding to the adult worm stage), animals were randomized into experimental groups (*n* = 5 per group). All treatments were administered by oral gavage. Infected mice were allocated into groups of five and treated with either PTL (400 mg/kg) or NLC-PTL (corresponding to 120 mg/kg of encapsulated PTL). An untreated group served as the control, while a separate group received blank NLC (NLC-B, 120 mg/kg). Praziquantel (PZQ; reference drug) was administered at 400 mg/kg. An infected untreated group was included as a negative control. The dose of 400 mg/kg was selected as it represents a standard reference dose widely used in experimental antischistosomal drug discovery studies, including for PZQ [1,30]. The lower dose used for NLC-PTL reflects both the improved delivery expected from nanoencapsulation and formulation constraints associated with high drug loading in lipid-based nanocarriers.

Ten days after treatment (day 59 post-infection), animals were euthanized. Adult worms were recovered by perfusion of the portal system, counted, and sexed. Therapeutic efficacy was evaluated based on the reduction in worm burden compared to the control group, as well as by analysis of the oogram pattern using a 10 mm fragment of the ascending colon and quantification of eggs per gram of feces (EPG) [9,30,36]. All animals completed the study, with no observed mortality during the experimental period.

#### 2.5.3. Ethical Approval

Animals were randomly assigned to the experimental groups according to a predefined randomization protocol, and euthanasia was performed in a randomized sequence. In vivo treatment allocation was balanced among the groups following established procedures [9,37,38]. The experimental design and reporting conformed to the ARRIVE guidelines recommended by the NC3Rs and complied with the Brazilian “Guidelines for the Care and Use of Laboratory Animals” (Law No. 11.794/2008). All procedures were approved by the Animal Ethics Committee of Guarulhos University, Guarulhos, São Paulo, Brazil (protocol CEUA 65/2024; Guarulhos University).

### 2.6. Statistical Analysis

Statistical analyses were carried out using GraphPad Prism 7.0 (GraphPad Software Inc., La Jolla, CA, USA). Prior to analysis, data were assessed for normality and homogeneity of variance to verify the assumptions for parametric testing. For the evaluation of in vivo data, differences between treatment groups and the control were assessed using a parametric Tukey’s multiple comparison test. The number of animals analyzed per group is indicated in the figure legends. Data are presented as mean ± standard deviation (SD), and individual data points are shown in the figures. Results were considered statistically significant when *p* < 0.05 [38].

## 3. Results

### 3.1. Isolation, Purification and Identification of PTL

**Figure 1:**
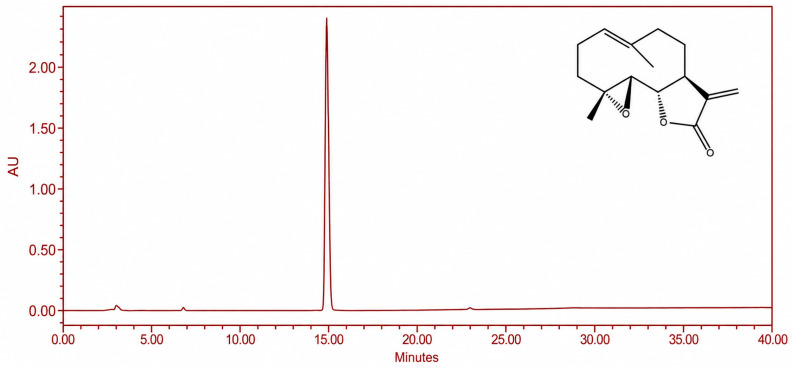
HPLC-DAD chromatogram (220 nm) and structure of PTL.

The extract of *T. parthenium* (TpE) was subjected to chromatographic fractionation to isolate PTL. As depicted in Figure 1, PTL was successfully isolated from TpE. This high purity exceeding 96% was confirmed through HPLC-DAD analysis (Figure 1), as well as through ^1^H and ^13^C NMR data (see Supplementary Figures S1 and S2). PTL was identified by ^1^H- and ^13^C-NMR data analysis in comparison to literature [13,39]. ^1^H NMR (CDCl_3_, 500 MHz): δ 1.210–1.273 (2H, td, J1 12.5 Hz and J2 5.5 Hz, H-3a), 1.27 (3H, s, H-15), 1.709 (3H, s, H-14), 1.727–1.757 (1H, td, J1 8.5 Hz and J2 2.0 Hz, H-8b), 2.121–2.19 (1H, s, H-2a, H-3b, H-8a, H-9a, overlapping peak), 2.121–2.191 (1H, m, H-9b), 2.390–2.452 (1H, td, J1 14.5 HZ and J2 5.0 Hz, H-2b), 2.756–2.780 (1H, dd, J1 8.5 Hz and J2 3.5Hz, H-7), 2.791–2.803 (1H, d, J 6.0 Hz, H-5), 3.856 (1H, t, J 8.5 Hz, H-6), 5.122–5.192 (1H, dd, J1 13.0 and J2 2.0 Hz, H-1), 5.615–5.622 (1H, d, J 3.0 Hz, H-13b), 6.325–6.333 (1H, d, J 4.0 Hz, H-13a). ^13^C NMR (CDCl3, 125 MHz): δ 17.10 (C-14), 17.42 (C-15), 24.29 (C-2), 30.80 (C-8), 36.50 (C-3), 41.36 (C-9), 47.82 (C-7), 61.70 (C-4), 66.55 (C-5), 82.60 (C-6), 121.41 (C-13), 125.43 (C-1), 134.74 (C-10), 139.38 (C-11), 169.42 (C-12).

### 3.2. Evaluation of Nanoparticles Containing PTL

#### 3.2.1. Particle Size, Polydispersity Index, Zeta Potential, and Encapsulation Efficiency

**Figure 2:**
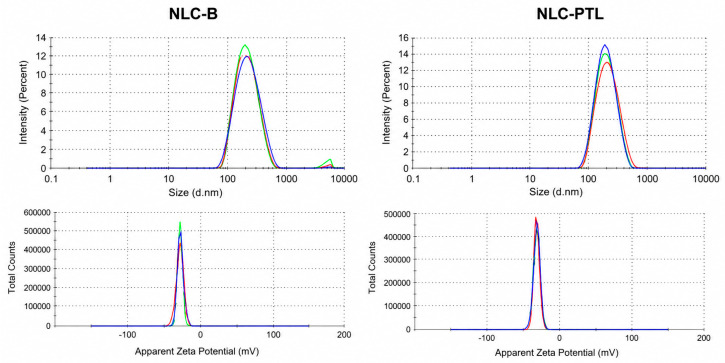
PS and ZP distributions by intensity for the developed NLC formulations (NLC-B and NLC-PTL).

The physicochemical characterization of the formulations is summarized in Table 2. NLC-B presented a PS of 196.3 ± 3.3 nm and a PdI of 0.184 ± 0.004, while the NLC-PTL formulation exhibited a PS of 173.6 ± 0.6 nm and a PdI of 0.114 ± 0.011. The ZP values were −35.2 ± 0.2 mV for NLC-B and −34.7 ± 0.4 mV for NLC-PTL. The pH values were 6.03 ± 0.01 for NLC-B and 8.40 ± 0.06 for NLC-PTL. The EE% of PTL in the NLC-PTL formulation was 77.98 ± 1.5%. The distribution of PS and ZP profiles by intensity of the developed formulations are presented in Figure 2.

#### 3.2.2. In Vitro Release Profile

**Figure 3:**
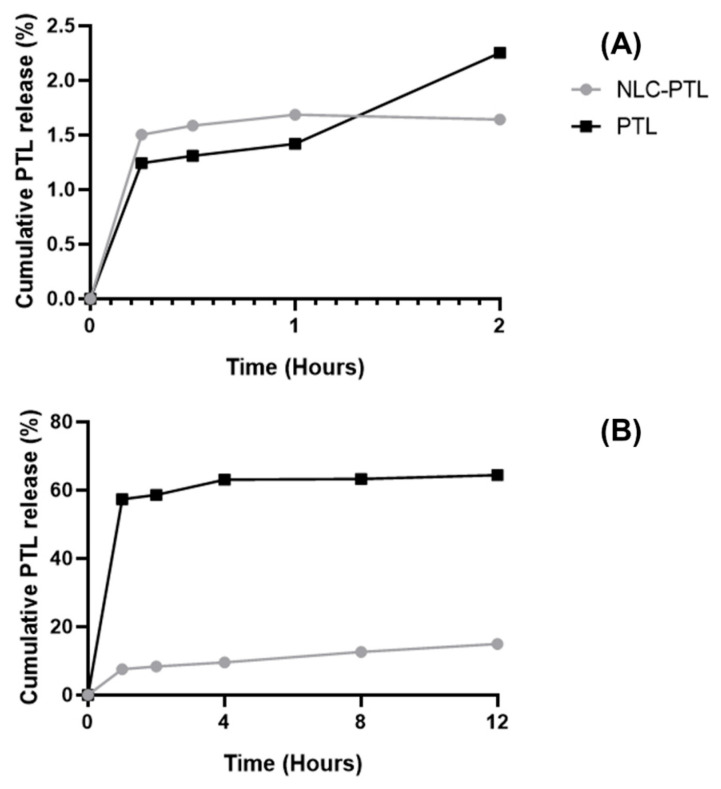
In vitro profiles of PTL-loaded NLC. Data represent mean ± SD from three independent experiments performed in triplicate. (**A**) receptor solution pH 1.2. (**B**) receptor solution pH 6.8. All points showed a statistically significant difference, *p* < 0.001, with the control group, in the (**B**) using the *t*-test and two-way ANOVA comparison test.

The in vitro release of NLC-PTL and free PTL was evaluated under simulated physiological conditions, as shown in Figure 3. The cumulative release profiles obtained at pH 1.2 (Figure 3A), over 2 h, and at pH 6.8 (Figure 3B), over 12 h, are presented. At pH 1.2, free PTL reached a release of 2.25% ± 0.016, while NLC-PTL showed a release of 1.64% ± 0.012. At pH 6.8, free PTL exhibited a cumulative release of 64.46% ± 0.14. In contrast, NLC-PTL showed an initial release of 7.56% ± 0.22 within the first hour, followed by a cumulative release of 14.13% ± 1.17 after 12 h. A statistically significant difference (*p* < 0.001) was observed between the cumulative release of PTL from the NLC-PTL formulation and free PTL at pH 6.8 (Figure 3B). Regarding the release kinetics, NLC-PTL showed the best fit to the Korsmeyer–Peppas model (R^2^ = 0.99; AIC = 1.70; *n* = 0.28) (Table 3).

#### 3.2.3. Colloidal Stability

After 90 days of storage at 4 °C, the NLC-PTL formulation showed PS, PdI, and ZP values of 175.5 ± 0.46 nm, 0.12 ± 0.01, and −31.30 ± 0.29 mV, respectively, with no significant differences compared to the initial values. The NLC-B formulation presented a ZP of −28.50 ± 1.93 mV, while particle size and PdI were 196.60 ± 2.08 nm and 0.19 ± 0.01, respectively. A statistically significant reduction in ZP was observed for NLC-B. The results obtained after 90 days of storage are presented in Supplementary Figure S3.

### 3.3. In Vivo Antischistosomal Effects of PTL and NLC-PTL Against S. mansoni

**Figure 4:**
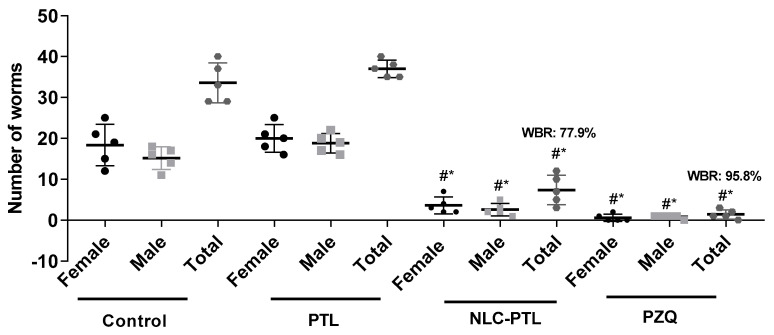
Effects of a single oral dose of PZQ (400 mg/kg), PTL (400 mg/kg), and NLC-PTL (corresponding to 120 mg/kg of encapsulated PTL) on worm burden in mice infected with 42-day-old *S. mansoni*. Worm burden was stratified according to sex (female, male, and total worms). Experimental groups included infected untreated controls and infected mice treated with PTL, NLC-PTL, or PZQ. Data are expressed as mean ± SD with individual values shown for each animal (*n* = 5 animals/group). Statistical analysis was performed using two-way ANOVA followed by Tukey’s multiple comparisons test. Significance levels: * *p* < 0.001 vs. control; # *p* < 0.001 vs. free PTL. WBR = worm burden reduction.

The therapeutic potential of PTL and NLC-PTL was evaluated in a murine schistosomiasis model. Oral treatment with free PTL (400 mg/kg) did not result in a significant reduction in worm burden compared with the untreated control group. In contrast, treatment with NLC-PTL (corresponding to 120 mg/kg of PTL) resulted in a significant total worm reduction of 77.9% (*p* < 0.001 vs. control) and showed a statistically significant reduction in worm burden compared with free PTL (*p* < 0.001). Likewise, the positive control, PZQ (400 mg/kg), achieved a 95.8% reduction in worm burden and differed significantly from free PTL (*p* < 0.001 vs. control; *p* < 0.001 vs. free PTL). No significant worm reduction was observed in mice treated with blank NLC (Figure 4).

**Figure 5:**
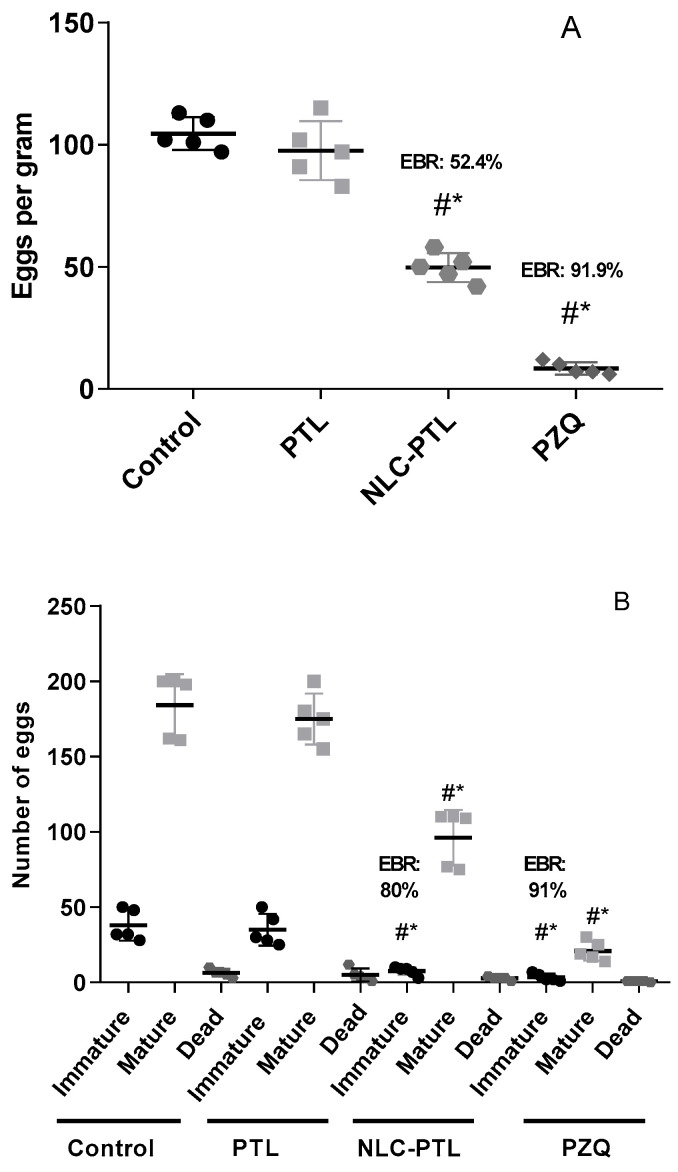
Effects of a single oral dose of PZQ (400 mg/kg), PTL (400 mg/kg), and NLC-PTL (corresponding to 120 mg/kg of encapsulated PTL) on egg burden and oogram patterns in mice infected with 42-day-old *S. mansoni*. (**A**) Eggs per gram of feces (EPG). (**B**) Oogram analysis showing immature, mature, and dead eggs. Experimental groups included infected untreated controls and infected mice treated with PTL, NLC-PTL, or PZQ. Data are expressed as mean ± SD with individual values shown for each animal (*n* = 5 animals/group). Statistical analysis was performed using two-way ANOVA followed by Tukey’s multiple comparisons test. Significance levels: * *p* < 0.001 vs. control; # *p* < 0.001 vs. free PTL. EBR = Egg Burden Reduction.

Also, analysis of fecal egg counts revealed reductions of 52.4% and 91.9% in mice treated with NLC-PTL and PZQ, respectively (* *p* < 0.001 vs. control; # *p* < 0.001 vs. free PTL) (Figure 5A). Furthermore, evaluation of the oogram patterns demonstrated substantial decreases in immature egg stages by 80.0% and 91.0% following treatment with NLC-PTL and PZQ, respectively (* *p* < 0.001 vs. control; # *p* < 0.001 vs. free PTL) (Figure 5B). In addition, free PTL (400 mg/kg, p.o.) and blank NLC did not show significant activity on stool egg load or egg developmental stages (oogram).

## 4. Discussion

The findings of this study indicate that nanoencapsulation significantly improves the in vivo antischistosomal efficacy of PTL. Although the free compound did not exhibit detectable antischistosomal activity, its delivery via NLC led to a substantial reduction in worm burden (77.9%) along with a pronounced decrease in egg production, underscoring the marked difference in efficacy between the two forms.

The physicochemical characterization of the developed formulations provides important insights into the influence of PTL incorporation on nanoparticle organization and performance. The reduction in PS observed after drug loading suggests that PTL interferes with lipid chain packing, promoting a less ordered and more compact internal structure. This behavior has been widely reported for lipid-based nanocarriers, in which drug–lipid interactions modify the crystalline arrangement of the lipid matrix, directly impacting nanoparticle size and structural organization [40]. Furthermore, the low PdI (<0.3) obtained for NLC-PTL indicates a homogeneous size distribution, which is a key factor for ensuring reproducibility, uniform drug distribution, and predictable biological performance [41].

The surface charge of the formulations, characterized by negative ZP values, can be attributed to the physicochemical composition of the system. In formulations stabilized with nonionic surfactants such as Pluronic^®^ F-127, negative charges are often associated with the partial ionization of fatty acids present in the lipid matrix, as well as the adsorption of hydroxyl ions at the oil–water interface [42,43]. The magnitude of these values suggests the presence of sufficient electrostatic repulsion to prevent particle aggregation, contributing to the overall colloidal stability of the system [44,45].

The pH variation observed between formulations with and without PTL provides additional evidence of interactions occurring within the system. While the slightly acidic pH of the blank formulation is consistent with dispersions stabilized by Pluronic^®^ F127, the increase in pH after PTL incorporation may be related to the presence of non-encapsulated drug molecules in the aqueous phase [46,47,48,49]. These pH changes may influence interfacial properties and contribute to maintaining dispersion stability by modulating electrostatic interactions between particles [50].

The high EE% obtained for NLC-PTL reflects the suitability of the lipid matrix to accommodate PTL. This result is consistent with the lipophilic nature of the compound, which favors its partitioning into the lipid phase during nanoparticle formation [47,51]. In addition, the combination of solid and liquid lipids likely contributes to a partially disordered internal structure, increasing the availability of accommodation sites and improving drug retention within the nanoparticles [27,52].

Specifically, although only 14.13% of PTL was released from NLC-PTL over 12 h under the in vitro conditions, this result may not directly reflect the in vivo behavior of the formulation following oral administration. In lipid-based nanosystems, dialysis-based release assays frequently underestimate drug availability due to the absence of physiological factors involved in gastrointestinal processing, including bile salts, digestive enzymes, lipid digestion, and nanoparticle–mucosa interactions [53,54]. Moreover, increasing evidence suggests that lipid nanoparticles may undergo partial uptake by the intestinal epithelium, enabling drug transport and absorption even in the absence of extensive prior drug release in the gastrointestinal lumen [55,56,57]. In this context, the substantial reduction in worm burden observed after treatment with NLC-PTL suggest that pharmacologically relevant amounts of PTL reached the biological target under in vivo conditions despite the restricted release profile observed in vitro. Collectively, these findings reinforce the complexity of establishing direct in vitro–in vivo correlations for nanocarrier-based systems [58].

To further characterize the release behavior, the experimental data were fitted to the Korsmeyer–Peppas model. According to this model, release exponent values lower than 0.43 for spherical systems are generally considered indicative of Fickian diffusion-controlled mechanisms [59,60]. In the present study, the release exponent obtained for NLC-PTL (*n* = 0.28) is therefore consistent with a predominantly diffusion-controlled release profile [61]. Nevertheless, considering the structural complexity and heterogeneity of lipid-based nanosystems, as well as the recognized limitations of the Korsmeyer–Peppas model in fully describing drug release from complex lipid matrices, these findings should be interpreted with caution. Indeed, very low *n* values may also reflect contributions from interfacial phenomena, surface erosion-related effects, or anomalous short-time release behavior that are not entirely captured by the classical diffusion-based interpretation [62].

Taken together, the stability results demonstrate that PTL incorporation contributes to maintaining the physicochemical properties of the system over time. The preservation of PS distribution and surface charge suggests that the internal organization of the lipid matrix remains stable during storage. In contrast, the changes observed in the blank formulation indicate that the absence of the drug may allow greater molecular rearrangement at the particle interface. These findings reinforce the role of drug-lipid interactions in stabilizing the nanostructured system and preserving its colloidal properties [63]. These results support the suitability of the developed NLC as a stable delivery platform and provide a foundation for evaluating their in vivo therapeutic performance.

Overall, these findings reinforce the concept that the performance of lipid-based nanosystems is intrinsically associated with the structural organization of the lipid matrix and with process-dependent formulation parameters. In addition, the low variability observed for PS, PdI, and ZP highlights the robustness and reproducibility of the selected preparation strategy for obtaining homogeneous nanosystems with consistent physicochemical properties. Such observations agree with classical studies on NLC systems, which established matrix heterogeneity and internal lipid organization as critical determinants of drug accommodation, release characteristics, physicochemical stability, and overall nanocarrier performance [45,64,65].

Furthermore, in vivo treatments with free PTL and NLC-PTL were conducted in mice with established *S. mansoni* infection. The dose of 400 mg/kg used for free PTL corresponds to a standard reference dose widely employed in experimental antischistosomal studies, including for praziquantel [1,35,66], whereas the lower dose selected for NLC-PTL (corresponding to 120 mg/kg of encapsulated PTL) reflects both the improved delivery expected from nanoencapsulation and formulation constraints associated with high drug loading in lipid-based nanocarriers. Following oral administration, free PTL produced no significant reduction in worm counts, whereas NLC-PTL treatment resulted in marked decreases in both worm burden and egg output compared with the control group. Moreover, NLC-PTL showed a statistically significant reduction in worm burden compared with free PTL (*p* < 0.001). Nevertheless, it is important to note that the study design did not include a matched-dose group of free PTL at 120 mg/kg. Therefore, it cannot be excluded that free PTL might exhibit partial efficacy at intermediate doses between 120 and 400 mg/kg. This limitation should be considered when interpreting the comparative performance of the nanoformulated and free drug.

Consistent with these observations, treatment with NLC-PTL led to a marked reduction in fecal egg output, whereas administration of free PTL did not significantly affect egg elimination or alter oogram profiles. Similar patterns have been reported for nanoformulated antiparasitic agents, in which improved delivery systems enhance reductions in egg burden compared with non-encapsulated compounds [30,67]. Importantly, oral administration of NLC-PTL, at a dose equivalent to 120 mg/kg of PTL, was not associated with mortality or overt behavioral or clinical signs of toxicity during the experimental period.

These findings are in agreement with previous studies suggesting that NLC systems may influence the in vivo performance of lipophilic compounds by modifying parameters related to drug release and gastrointestinal interaction [68]. In this context, NLC-based formulations of PZQ and other compounds have been reported to show improved antiparasitic activity when compared with their non-encapsulated counterparts [11,68].

The present results indicate that nanoencapsulation may influence the in vivo activity of PTL. However, the mechanisms underlying this effect remain unclear. Although changes in apparent solubility, physicochemical stability, or gastrointestinal interaction may have contributed to the enhanced efficacy observed for NLC-PTL, these possibilities remain speculative, since pharmacokinetic and biodistribution analyses were not performed in the present study. Therefore, the present data does not allow conclusions regarding systemic exposure, tissue distribution, or availability of PTL at the parasite site. In addition, other factors related to the formulation itself, including carrier-associated or local gastrointestinal effects, cannot be excluded.

Despite these promising results, some limitations should be acknowledged. The study did not include pharmacokinetic or biodistribution analyses, which will be important in future studies to clarify the mechanisms associated with the improved in vivo efficacy of NLC-PTL. In addition, toxicity assessment was limited to observational parameters, and further studies are required to establish the safety profile of the formulation. Moreover, the in vivo experiments were conducted with a relatively small number of animals per group (*n* = 5), which is consistent with standard practice in preliminary antischistosomal drug discovery studies but may limit statistical power [69,70]. This aspect has been mitigated by the inclusion of individual data points and variability measures, although it should still be considered when interpreting the results.

Overall, these results suggest that nanoencapsulation substantially improves the in vivo antischistosomal efficacy of PTL. Although the underlying mechanisms remain to be fully elucidated, the findings support the potential of NLC as a promising strategy for enhancing the in vivo activity of poorly water-soluble natural compounds. Future studies involving plasma concentration time analysis, tissue biodistribution, and exposure profiling will be important to clarify the mechanisms associated with the improved efficacy of NLC-PTL.

## 5. Conclusions

In this study, PTL was isolated from *T. parthenium* extract and successfully incorporated into NLC. The formulation exhibited suitable physicochemical properties, including nanoscale PS, favorable surface charge, high EE%, homogeneous PdI, controlled drug release, and physical stability for 90 days. In vivo, orally administered NLC-PTL demonstrated greater efficacy against *S. mansoni* than non-encapsulated PTL, resulting in marked reductions in worm burden and egg output. These findings suggest that NLC improves the in vivo antischistosomal efficacy of PTL and highlight the potential of this formulation strategy for schistosomiasis treatment. Future studies should further investigate the pharmacokinetic and biodistribution profiles of NLC-PTL, as well as the mechanisms underlying its enhanced antiparasitic activity. More broadly, this work supports the continued exploration of nanotechnology-based drug delivery systems for schistosomiasis and other neglected parasitic diseases.

## Figures and Tables

**Table 1:** Composition of drug-free (NLC-B) and PTL-loaded nanostructured lipid carriers (NLC-PTL).

Composition % (*w*/*v*)				
	PB	M812	PF-127	PTL
NLC-B	3.9	3.0	1.8	-
NLC-PTL	3.9	3.0	1.8	0.1

**Table 2:** Physicochemical properties of NLC-B and NLC-PTL.

Physicochemical Properties					
	PS (nm)	PdI	ZP (mV)	EE (%)	pH
**NLC-B**	196.3 ± 3.3	0.184 ± 0.004	−35.2 ± 0.2	-	6.03 ± 0.01
**NLC-PTL**	173.6 ± 0.6	0.114 ± 0.011	−34.7 ± 0.4	77.98 ± 1.5	8.40 ± 0.06

Data are expressed as the mean ± S.D. (*n* = 3).

**Table 3:** Release kinetics model fitting parameters for NLC-PTL.

	NLC-PTL pH 6.8	
Kinetics model	R^2^	AIC
Zero order (K_0_)	0.24	29.69
First order (K_1_)	0.30	29.21
Higuchi (K_H_)	0.88	18.36
Korsmeyer–Peppas (K_KP_) (*n* value)	0.99 (0.28)	1.70

## Data Availability

The original contributions presented in this study are included in the article/Supplementary Material. Further inquiries can be directed to the corresponding author.

## Supplementary Materials

The following supporting information can be downloaded at: https://www.mdpi.com/article/10.3390/pharmaceutics18060694/s1, Figure S1. Parthenolide ^1^H NMR (CDCl_3_, 500 MHz). Figure S2. Parthenolide ^13^C NMR (CDCl_3_, 125 MHz) and DEPT 135. Figure S3. Evaluation of PS, PdI and ZP stability over 90 days of storage.

## Abbreviations

The following abbreviations are used in this manuscript:

PZQ	Praziquantel
PTL	Parthenolide
NLC	Nanostructured Lipid Carriers
NLC-PTL	Nanostructured Lipid Carriers with Parthenolide
NLC-B	Drug-free NLC
PS	Particle Size
PdI	Polydispersity Index
ZP	Zeta Potential
EE%	Encapsulation Efficiency
