# Supplementary material for: Nanostructured Lipid Carriers Enable In Vivo Efficacy of Parthenolide in Schistosoma mansoni Infection
Source: Pharmaceutics. 2026 Jun 3;18(6):694. doi: 10.3390/pharmaceutics18060694 (PMC13306080; doi:10.3390/pharmaceutics18060694)
Supplement: Supplementary file 1 [file pharmaceutics-18-00694-s001.zip › pharmaceutics-4315412-supplementary.pdf]

pharmaceutics

MDPI

SUPPORTING INFORMATION

Nanostructured Lipid Carriers Enable In Vivo Efficacy of Parthenolide in *Schistosoma mansoni* Infection

José Márcio Fernandes da Silva [1], Dominique Mesquita e Silva [1], Danilo de Souza Costa [1], Monique C. Amaro [2], Rayssa A. Cajas [2], Josué de Moraes [2,3], Guilherme Diniz Tavares [1] and Ademar Alves Da Silva Filho [1,*]

1 Departamento de Ciências Farmacêuticas, Faculdade de Farmácia, Universidade Federal de Juiz de Fora, R. José Lourenço Kelmer s/n, Campus Universitário, Juiz de Fora 36036-900, MG, Brazil; jmf_farm@yahoo.com.br (J.M.F.d.S.); dominiquefarmacia@gmail.com (D.M.e.S.); scdanilo@gmail.com (D.d.S.C.); guilherme.tavares@ufjf.br (G.D.T.)
2 Núcleo de Pesquisa em Doenças Negligenciadas, Universidade Guarulhos, Guarulhos 07023-070, SP, Brazil; moniqueamaronpdn@gmail.com (M.C.A.); rayssacajas@gmail.com (R.A.C.); moraesnpdn@gmail.com (J.d.M.)
3 Núcleo de Pesquisa em Doenças Negligenciadas, Instituto Científico e Tecnológico, Universidade Brasil, São Paulo 08230-030, SP, Brazil
* Correspondence: ademar.alves@ufjf.br; Tel.: +55-32-21023893

 https://doi.org/10.3390/pharmaceutics18060694

Supplementary Figure S1. Parthenolide ^{1}H NMR ($CDCl_3$, 500 MHz).

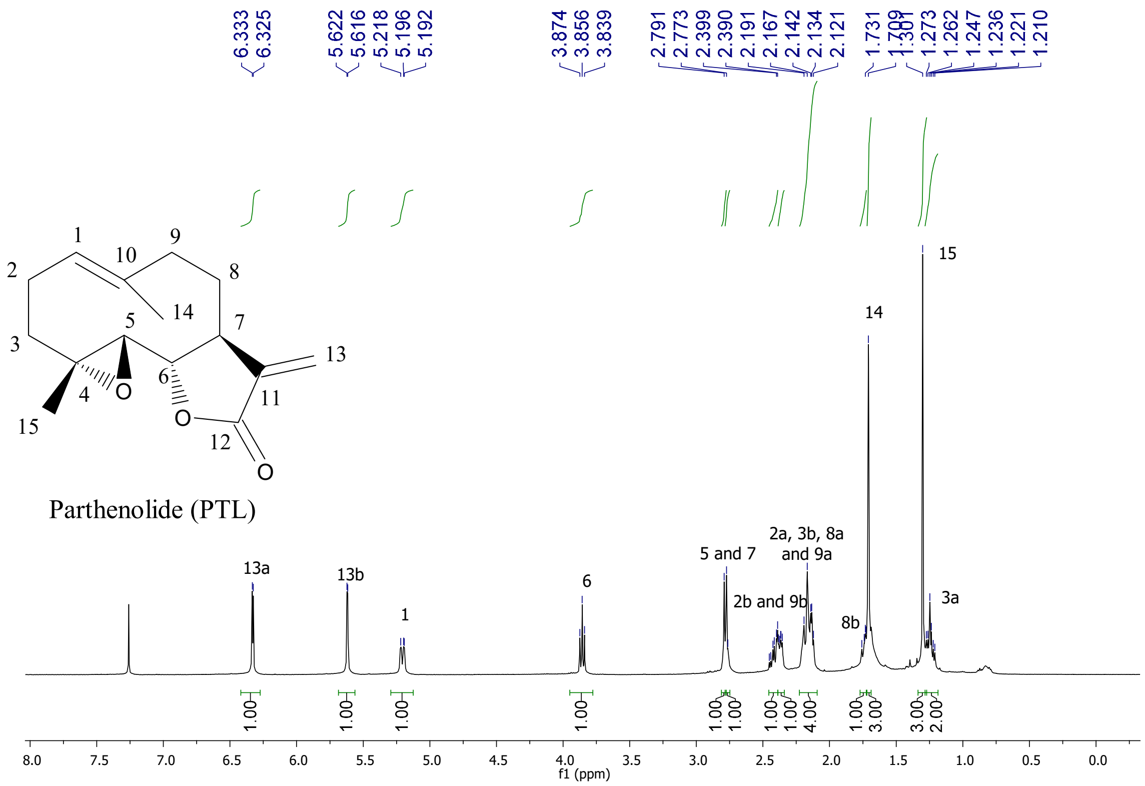

https://doi.org/10.3390/pharmaceutics18060694

Supplementary Figure S2. Parthenolide ^{13}C NMR ($CDCl_3$, 125 MHz) and DEPT 135.

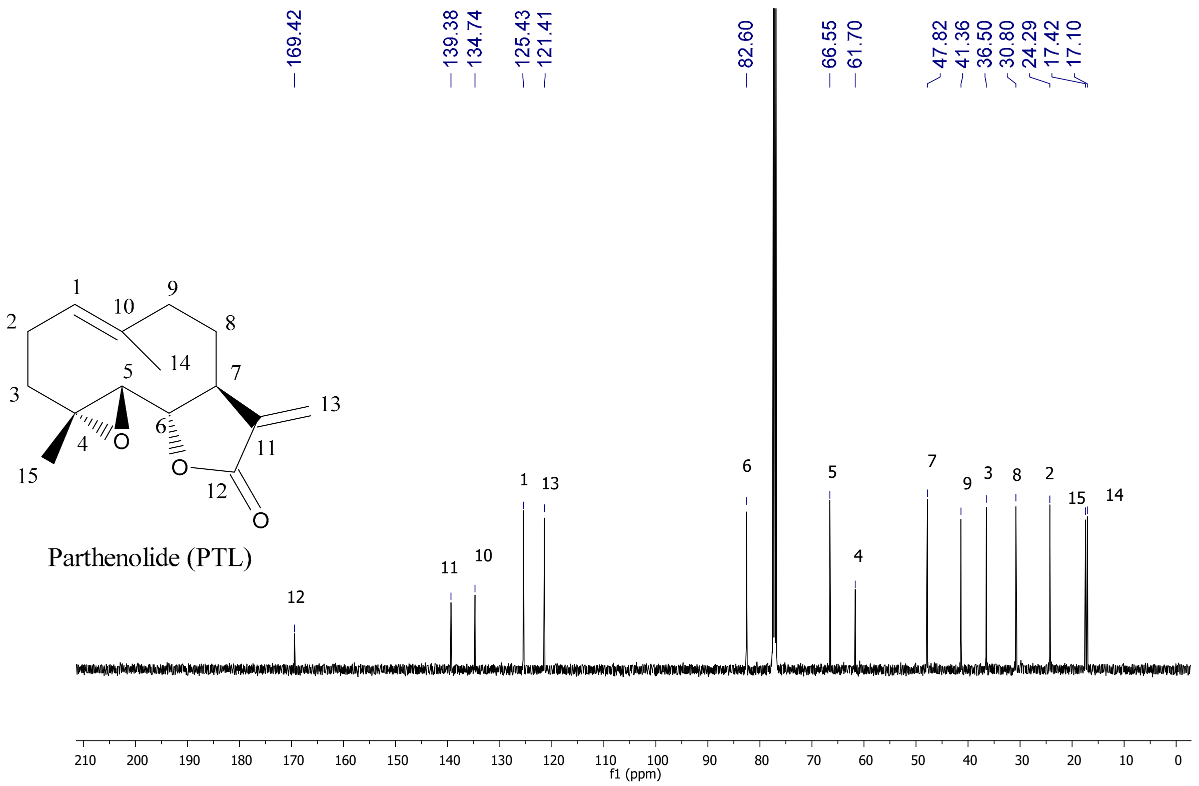

Supplementary Figure S3. Evaluation of PS, PdI and ZP stability over 90 days of storage. Measurements were performed at 0, 30, 60, and 90 days. Data represent mean ± SD from three independent experiments performed in triplicate. *P < 0.001 compared to the day 0 (control) group using Dunnett's multiple comparison test.

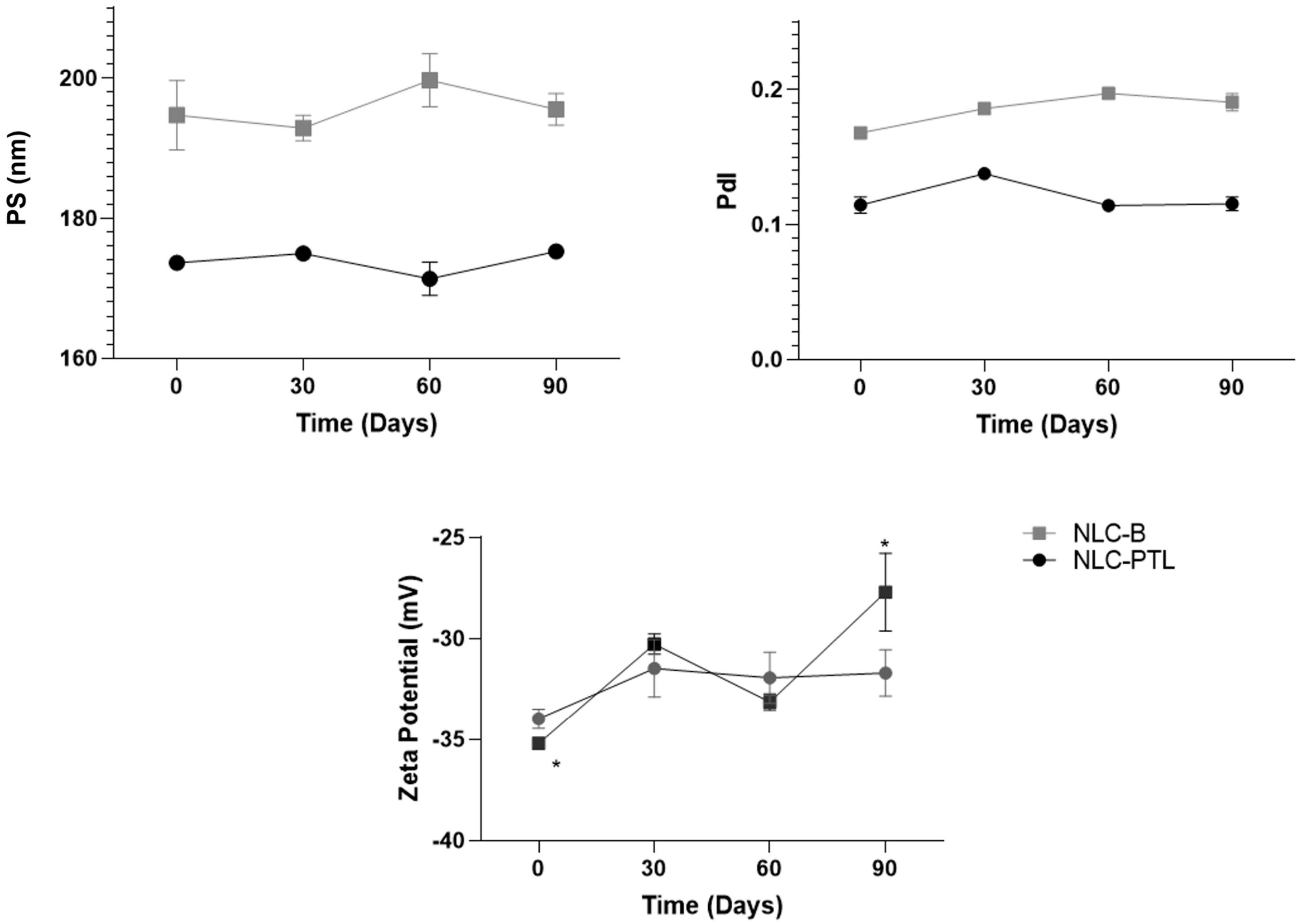

https://doi.org/10.3390/pharmaceutics18060694
